# Reversible data hiding in encrypted images with multi-prediction and adaptive huffman encoding

**DOI:** 10.1038/s41598-023-50186-1

**Published:** 2023-12-29

**Authors:** Hua Ren, Guang-rong Bai, Tong-tong Chen, Zhen Yue, Ru-yong Ren

**Affiliations:** 1 College of Computer and Information Engineering, Henan Normal University, Xinxiang, 453007 Henan China; 2 Key Laboratory of Artificial Intelligence and Personalized Learning in Education of Henan Province, Xinxiang, Henan China; 3 Faculty of Education, Henan Normal University, Xinxiang, China; 4 Beijing Key Lab of Intelligent Telecommunication Software and Multimedia, School of Computer, Beijing University of Posts and Telecommunications, Beijing, 100876 China

**Keywords:** Information technology, Computer science

## Abstract

With the rapid development of multimedia technology and the massive accumulation of user data, a huge amount of data is rapidly generated and shared over the network, while the problems of inappropriate data access and abuse persist. Reversible data hiding in encrypted images (RDHEI) is a privacy-preserving method that embeds protected data in an encrypted domain and accurately extracts the embedded data without affecting the original content. However, the amount of embedded data has been one of the major limitations in the performance and application of RDHEI. Currently, the main approaches to improve the capacity of RDHEI are either to increase the overall capacity or to reduce the length of the auxiliary information. In this paper, we propose a novel RDHEI scheme based on multi-prediction and adaptive Huffman encoding. To increase the overall capacity, we propose a multi-prediction, called MED+GAP predictor, to generate the label map data of non-reference pixels prior to image encryption. Then, an adaptive Huffman coding is designed to compress the generated labels in order to reduce the embedding length of the auxiliary information used for the extraction and recovery. Experiments show that the proposed method with MED+GAP predictor and adaptive Huffman coding improves 0.052 bpp, 0.023 bpp, and 0.047 bpp on average over the other state-of-the-art methods on the BOSSBase, BOWS-2, and UCID datasets, respectively, while maintaining security and reversibility.

## Introduction

Nowadays, an increasing number of individuals are uploading their private images to cloud platforms because of the convenient access to shared resources provided by cloud storage devices. Although this approach reduces costs associated with storage and maintenance, there are still security and data management challenges that arise from the accumulation of user data. Data hiding is an effective technique for protecting privacy, as it involves embedding data in multimedia content in a subtly modified manner and extracting the embedded data without any errors. As a result, cloud managers do not need to allocate additional space for managing user data. In order to recover the carrier content losslessly when extracting the data, reversible data hiding (RDH) methods have been further developed. In recent decades, RDH methods such as difference expansion (DE)^1^, histogram shifting (HS)^2^, prediction error expansion (PEE)^3^ and pixel value ordering (PVO)^4^ have received increasing interest and have been widely applied in military communications, medical diagnosis and law forensics.

When the content of a multimedia carrier needs to be protected, the carrier can first be encrypted and then secret data can be embedded in the encrypted content, which is also known as reversible data hiding in encrypted images (RDHEI)^5,6,14^. It is an emerging research hotspot that allows reversible hiding in encrypted data and lossless recovery of the original plaintext carrier, involving three parties: the content-owner, the data-hider, and the receiver. The content-owner encrypts the original image while preserving some of the pixel redundancy of the carrier image, the data-hider embeds secret data based on the preserved redundancy, and the receiver holding the corresponding keys is able to accurately extract the secret data or recover the original image without loss. At present, the existing RDHEI schemes can be roughly divided into two main categories based on the order of image encryption and reserved embedding space: vacating room after encryption (VRAE) and reserving room before encryption (RRBE).

**Figure 1:**
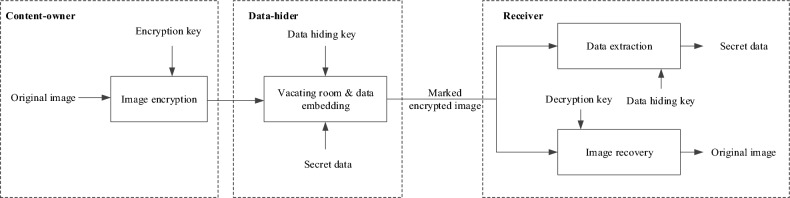
The VRAE framework.

The VRAE technique is designed to embed secret data within an encrypted image. Figure 1 illustrates the framework. The data-hider vacates room from the encrypted image and embeds secret data into the vacated room based on a data hiding key. In theory, this process is challenging because encryption increases the entropy of the carrier and reduces available redundancy space. However, VRAE-based RDHEI research has gained significant attention from various related studies^7–19^. In^7^, the original image was encrypted using a pseudorandom stream cipher, and one bit of the secret data was embedded by flipping the three least significant bits (LSBs) of all the pixels in a selected block. This work was the first to provide a theoretical validation of embedding secret data in the encrypted domain. However, since the smoothness of blocks is taken into account in the extraction phase, extraction errors can occur if inappropriate blocks are selected. Later, edge matching^8^ and full embedding^9^ mechanisms were proposed one after another. Unfortunately, all these mechanisms share a common problem: image decryption must be performed before the data can be extracted. To solve this issue, Zhang^10^ further compressed the LSBs of the encrypted image and embedded the secret data directly into the vacated LSBs to perform data extraction and image recovery operations separately. To increase the capacity, Liu and Pun^11^ proposed a redundant space transfer scheme that introduced a special encryption operation to redundantly transfer the plaintext to the ciphertext. Subsequently, Qin et al.^12^ provided an improved version for different types of encrypted binary blocks to achieve embedding of secret data using sparse matrix coding. Yi and Zhou^13^ developed a parametric binary tree labelling method and applied it to the VRAE scheme, achieving average embedding rates of 1.9656 bpp and 1.8808 bpp on the BOSSbass and BOW-2 datasets. Fu et al.^14^ used Huffman coding to compress the most significant bit (MSB) of the high bit plane of the embeddable block, embedding the auxiliary information, Huffman coding, and the secret data in the vacated space, and achieving a high embedding capacity. Wang et al.^15^ used an adaptive Huffman coding technique to compress the MSB bit planes. By preserving the auxiliary and encoded information, the carrier image can be recovered losslessly after data extraction. Zhou and Chen^16^ proposed a novel VRAE-based algorithm that combines block classification and multi-layer processing to fully explore the redundant space.

**Figure 2:**
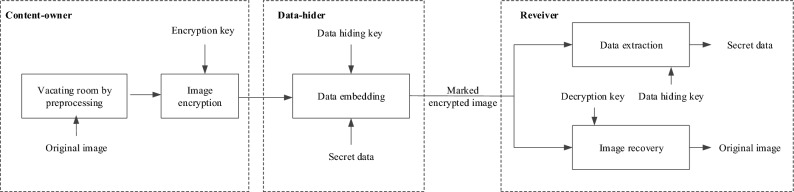
The RRBE framework.

In contrast, RRBE research requires a pre-processing operation before image encryption to free the embedding space, or more precisely, to create space in the plaintext domain^20–32^, as shown in Fig. 2. Ma et al.^20^ proposed the first RRBE solution to address capacity and visual quality issues in VRAE. Building on this, Malik et al.^21^ developed a prediction error method to pre-process images to create the redundant space. Specific locations of embedded data were captured based on bitmap information and secret data was embedded the captured locations using MSB bit planes. To better exploit the correlation between adjacent pixels, Cao et al.^22^ considered a block-level sparse representation in the hiding of secret data. Yin et al.^23^ used pixel prediction and multi-MSB rearrangement to embed more secret data in the reserved space. Since the rearrangement involves a large amount of auxiliary information, this mechanism uses lossless arithmetic coding to compress the auxiliary information, and then embeds the compressed information together with the secret data into the reserved space. Yin et al.^32^ used multi-MSB prediction and Huffman coding for the original image to perform a bit substitution operation on the embeddable pixels based on the auxiliary information. Non-reference pixels in the original image were predicted using a MED detector to generate their predicted values, and nine predefined Huffman codes 00,01,100,101,1100,1101,1110,11110,11111 were used to encode the generated prediction values to yield the label map data.

However, there are two issues with the scheme^32^. One is that the label map data generated from different images is compressed using predefined nine variable-length encodings, which may not be the most optimal method. Alternatively, finding the most appropriate nine variable-length encoding mapping rules for different images could reduce the length of the binary label map, thereby improving the embeddability. The other is that the MED median edge detector only uses the three pixels surrounding the current pixel to make the prediction, without sufficiently considering the effect of the other neighbouring pixels, which can lead to larger prediction errors. A larger error can lead to a reduction in the number of embedding bits that can be carried by the pixel label. Therefore, replacing a single prediction with a more accurate prediction algorithm that improves the prediction accuracy has the potential to further improve the embeddability. In view of this, the contributions of the proposed method are summarized as follows.

1. We propose a multi-prediction method to generate the predicted values. The original pixels are predicted using different predictors to fully exploit the influence of the other neighbouring pixels. This results in more embeddable bits of non-reference pixels, thereby creating more redundancy space.
2. We design an adaptive Huffman encoding method to encode the generated prediction values. The prediction values are adaptively encoded instead of using predefined encoding rules. The selection of different images can lead to different encoding rules. These encoding rules can adapt to the image characteristics and thus improve the embedding capacity.
3. We conduct a wide range of experiments, including single-prediction/multi-prediction and non-adaptive /adaptive encoding, to comprehensively analyze and verify the effectiveness of the proposed method. Experimental results show that the proposed method improves 0.052 bpp, 0.023 bpp, and 0.047 bpp on average compared to other state-of-the-art methods on the BOSSBase, BOWS-2, and UCID datasets, while maintaining security and reversibility.

## Predictors

**Figure 3:**
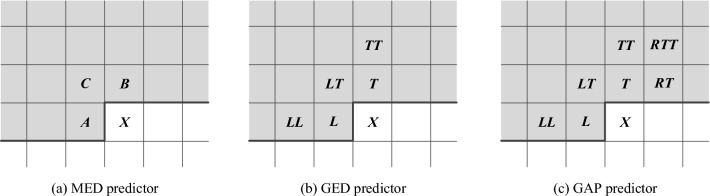
The prediction of the target pixel X based on different predictors. (**a**) MED predictor; (**b**) GED predictor; (**c**) GAP predictor.

In this section, we elaborate three predictor strategies, including median edge detector (MED), gradient edge detection predictor (GED), and gradient adjusted predictor (GAP). Figure 3 shows the prediction of the target pixel *X* based on different predictors. The MED predictor is a pixel prediction method that predicts the target pixel based on three neighbors *A*, *B* and *C*, as shown in Fig. 1a. The principle is that if there is a vertical edge to the left of the target position, then the target pixel value should be close to *B*; if there is a horizontal edge above the target position, then the current pixel value should be close to *A*; if there is no edge, then the current pixel value should be close to $B+A-C$, which is the median of the three pixels. The detailed MED prediction is formulated in the following Eq. (1).

1 $$\begin{aligned} {{P_X} = \left\{ \begin{array}{l} \begin{array}{*{20}{c}} {\max (B,A),}&{}{C \le \min (B,A)} \end{array}\\ \begin{array}{*{20}{c}} {\min (B,A),}&{}{C \ge \max (B,A)} \end{array}\\ \begin{array}{*{20}{c}} {B + A - C,}&{}{others} \end{array} \end{array} \right. } \end{aligned}$$

The GED predictor is another pixel prediction method that uses five neigbbors to estimate the local gradient to predict the target pixel. Figure 3b shows the GED prediction, where the shaded pixels in Fig. 3b are used to predict the target pixel *X*. The GED prediction involves three main elements, namely the horizontal gradient, the vertical gradient and the threshold. By comparing the relationship between these three elements, it is decided which pixels will ultimately be involved in the prediction of the current pixel *X*. Assuming that the horizontal gradient is denoted as $g_h$, the vertical gradient is denoted as $g_v$, and the threshold is denoted as $T_1$, the detailed GED prediction process is formulated in the following Eq. (2).

2 $$\begin{aligned} {{P_X} = \left\{ \begin{array}{l} \begin{array}{*{20}{c}} {L,}&{}{{g_v} - {g_h} > {T_1}} \end{array}\\ \begin{array}{*{20}{c}} {T,}&{}{{g_v} - {g_h} < - {T_1}} \end{array}\\ \begin{array}{*{20}{c}} {3(L + T)/8 + (LT + LL + TT)/12,}&{}{others} \end{array} \end{array} \right. } \end{aligned}$$

Where the horizontal gradient is computed as ${g_h} = |LL - L| + |LT - T|$ and the vertical gradient is computed as ${g_v} = |LT - L| + |TT - T|$.

The GAP predictor takes into account seven neighbors for the prediction of the target pixel *X*. Figure 3c shows the GAP prediction, and the shaded pixels are used to participate in the prediction process. Assuming that the thresholds are denoted as $T_2$, $T_3$ and $T_4$, the vertical gradient is denoted as $g_h$ and the horizontal gradient is denoted as $g_v$, we can compute the predict pixel *X* according to Eq. (3) and Eq. (4).

3 $$\begin{aligned} {{P_X} = \left\{ \begin{array}{l} \begin{array}{*{20}{c}} {L,}&{}{{g_v} - {g_h} > {T_2}} \end{array}\\ \begin{array}{*{20}{c}} {T,}&{}{{g_v} - {g_h} < - {T_2}} \end{array}\\ \begin{array}{*{20}{c}} {P,}&{}{others} \end{array} \end{array} \right. } \end{aligned}$$

The intermediate variable *P* is defined as follows:

4 $$\begin{aligned} {P = \left\{ \begin{array}{l} \begin{array}{*{20}{c}} {(X + L)/2,}&{}{{g_v} - {g_h}> {T_3}} \end{array}\\ \begin{array}{*{20}{c}} {(3X + L)/4,}&{}{{g_v} - {g_h} > {T_4}} \end{array}\\ \begin{array}{*{20}{c}} {(X + T)/2,}&{}{{g_v} - {g_h}< - {T_3}} \end{array}\\ \begin{array}{*{20}{c}} {(3X + T)/4,}&{}{{g_v} - {g_h} < - {T_4}} \end{array}\\ \begin{array}{*{20}{c}} {(L + T)/2 + (RT - LT)/4,}&{}{others} \end{array} \end{array} \right. } \end{aligned}$$

Where the horizontal gradient is computed as ${g_h} = |L - LL| + |T - LT| + |T - RT|$ and the vertical gradient is computed as ${g_v} = |L - LT| + |T - TT| + |RT - RTT|$. Based on the prediction accuracy, the thresholds are set to ${T_2} = 80$, ${T_3} = 32$ and ${T_4} = 8$^37^.

## The proposed method

**Figure 4:**
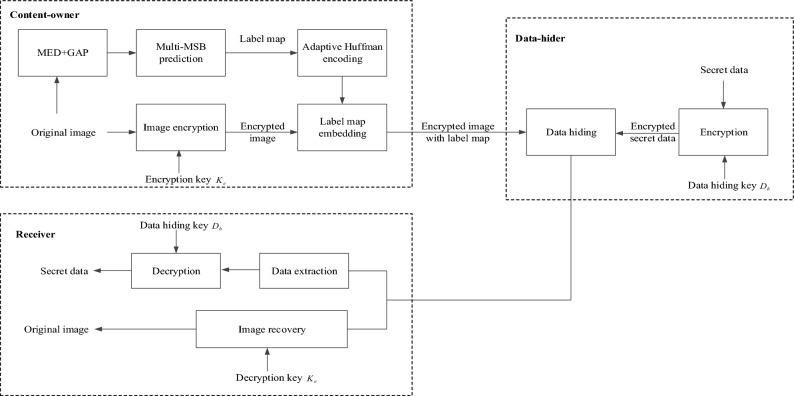
The framework of the proposed method.

Figure 4 illustrates the framework of the proposed method, from which we can identify three processes: pre-processing and image encryption by the content-owner, data embedding by the data-hider, and data extraction and image decryption by the receiver. Prior to image encryption, the content-owner first uses the MED+GAP predictor to compute a label map of non-reference pixels in the original image, and then encodes the generated label map using adaptive Huffman coding and embeds it in the encrypted image. After obtaining the label map, the data-hider can embed the secret data into the encrypted non-reference pixels using adaptive multi-MSB replacement. Finally, data extraction and image recovery can be performed for a receiver with the data hiding key and the image encryption key, while the extracted data and the recovered image are error-free.

We then give a detailed description of label map generation, image encryption, adaptive Huffman coding, label map embedding, data embedding, and data extraction and image recovery.

### Label map generation

In the label map generation process in the literature^32^, only three pixels around the current non-reference pixel are used to generate labels for non-reference pixels, but the influence of the other adjacent pixels is not fully considered. In contrast, GAP can achieve higher prediction accuracy after using seven adjacent pixels and three thresholds to predict the current non-reference pixel. However, when using GAP, only non-reference pixels with seven adjacent pixels can be predicted, which means that the reference pixels will change from the first row and column to the first two rows and columns. An increase in the number of reference pixels means a decrease in the number of non-reference pixels, which in turn reduces the embedding capacity. In this paper, we still consider only the first row and column as reference pixels, while pixels without enough adjacent pixels are predicted with MED and the rest of the pixels are predicted with GAP. This ensures the number of embeddable pixels and improves the accuracy of the prediction.

**Figure 5:**
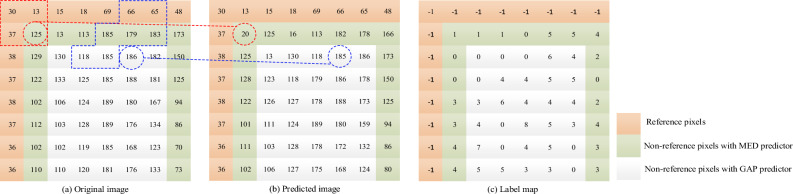
The proposed prediction process. (**a**) The original image; (**b**) The predicted image (**c**) The generated label map.

**Figure 6:**
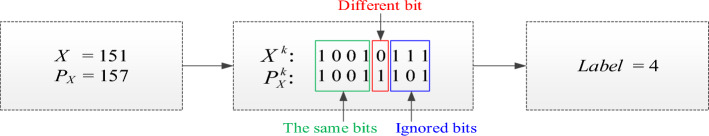
An example of pixel labelling.

Figure 5 shows a schematic of the designed MED+GAP predictor. In the figure, red and blue refer to the MED predictor and the GAP predictor respectively. The dashed circles in Fig. 5a indicate the target pixels, while those in Fig. 5b indicate the predicted pixels. The other two dashed areas refer to the neighbors of the target pixels. As shown in Fig. 5, the first row and column of the entire image content are reference pixels, while all other pixels are non-reference pixels. The predictions of all non-reference pixels are made by using the MED+GAP predictor together. The MED predictor with low pixel involvement is used to predict pixels that are close to both reference pixel and right border of the image. On the other hand, GAP predictor with high pixel involvement is used to predict remaining non-reference pixels. This approach generates the predicted values for all non-reference pixels. In order to illustrate our prediction process, We will take target pixel 125 (predicted by the MED predictor) and target pixel 186 (predicted by GAP predictor) as examples. For the target pixel 125, we can calculate the predicted value as $P_{125} = B+A-C=13+37-30=20$ according to the MED predictor in Eq. (2). The result is shown in the dashed circle in Fig. 5b. For the target pixel 186, we first calculate ${g_h} = |L - LL| + |T - LT| + |T - RT|=|185 - 79|+|179 - 185|+|179 - 183|=77$ and ${g_v} = |L - LT| + |T - TT| + |RT - RTT| = |185- 185| + |179 - 66| + |183 - 65| = 231$. Since the threshold $T_2 = 80$, we can calculate the predicted value as $P_{186} = L = 185$ according to Eq. (3), as shown in Fig. 5b. Finally, the label map is calculated by sequentially comparing the binary bits between the original image and the predicted image, and this process has been shown in Fig. 6. The label ‘-1’ in Fig. 5c indicates that all reference pixels are not involved in the labeling.

For all non-reference pixels *X*(*i*, *j*), both *X*(*i*, *j*) and the corresponding predicted value $P_X(i,j)$ are transformed into a binary sequence of eight-bit length.

5 $$\begin{aligned} {\begin{array}{*{20}{c}} {{X^k}(i,j) = \frac{{X(i,j)\bmod {2^{9 - k}}}}{{{2^{8 - k}}}},}&{1 \le k \le 8,1< i \le M,1 < j \le N} \end{array}} \end{aligned}$$

Where the *k*-th MSB bit of the 8-bit binary sequence of the current pixel *X*(*i*, *j*) is denoted as ${X^k}(i,j)$, and the parameters *M* and *N* are the row and column number of an image.

Figure 6 shows an example of pixel labelling. From Fig. 6, we can see that each bit of ${X^k}(i,j)$ and $P_X^k(i,j)$ is compared sequentially from MSB to LSB until one bit is different and the label value of the current pixel is equal to the length of its identical bits. If the label value is *t* ( $t \ne 8$), the $t + 1$ bit can be embedded because the first *t*-MSB of the original pixel is equal to its predicted value, and the ${(t + 1)^{th}}$ MSB can be obtained by negating the value at the position corresponding to its predicted value. All non-reference pixels in the image can be scanned by the above method to obtain the label map of the original image.

### Image encryption

In the whole image encryption process, we encrypt each pixel of the image using the stream cipher method. First, the key ${K_e}$ is determined, then a pseudo-random matrix *R* of size $M \times N$ is generated by ${K_e}$. Next, the current pixel *X*(*i*, *j*) and its corresponding pseudo-random value *R*(*i*, *j*) are converted into an 8-bit binary sequence ${X^k}(i,j)$ and ${R^k}(i,j)$ (${1 \le k \le 8}$) respectively, and they are encrypted by an exclusive operation in Eq. (6).

6 $$\begin{aligned} {\begin{array}{*{20}{c}} {X_e^k(i,j) = {X^k}(i,j) \oplus {R^k}(i,j),}&{1 \le k \le 8} \end{array}} \end{aligned}$$

where $X_e^k(i,j)$ is the encrypted binary sequence. We convert the encrypted sequence to the decimal value according to Eq. (7), and obtain the encrypted pixel $X_e(i,j)$.

7 $$\begin{aligned} {\begin{array}{*{20}{c}} {{X_e}(i,j) = \sum \limits _{k = 1}^8 {X_e^k(i,j) \times {2^{8 - k}},} }&{1 \le k \le 8} \end{array}} \end{aligned}$$

### Adaptive Huffman coding

Based on the generated label map, the embeddable bits for each non-reference pixel are known. However, the label map must be embedded into the embeddable bits of the non-reference pixels to ensure reversible embedding and extraction of the secret data. If the label map is embedded directly into the embeddable bits, the amount of space available for the embedding of the secret data is directly reduced. Therefore, this section considers lossless compression of the label map based on an adaptive Huffman encoding.

Huffman coding is a variable length encoding that constructs the shortest codeword based on the probability of a character occurring and achieves lossless data compression. For a given character set and probability distribution, the encoding produces the shortest codeword sequence on average. Specifically, a binary tree is used to represent the correspondence between characters and encodings, where the leaf nodes of the tree are characters and the branches of the tree are encodings, one branch being 0 and the other 1. The encoding results are not unique because (1) the Huffman tree construction when two symbols have the same probability is not unique, and (2) the choice of branches 0 and 1 is not fixed during the encoding process; it is possible to specify the left branch as 0 or the right branch as 0. These two reasons lead to the possibility of different encoding results. However, characters with a high probability of occurrence are specified as shorter codewords, while those with a low probability are specified as longer codewords. In view of this, we design an adaptive method to encode the generated label map. The detailed algorithm for constructing the Huffman tree is described in Algorithm 1.

**Algorithm 1:**
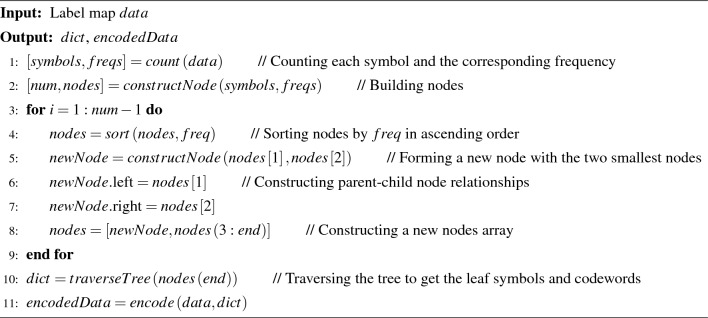
Adaptive Huffman encoding

The generated label map is converted into a binary sequence according to the mapping rules between the encoded label and the codeword, and the length of the binary sequence of the label map can be calculated by Eq. (8).

8 $$\begin{aligned} {\begin{array}{*{20}{c}} {L\mathrm{{en}} = \sum \limits _{t = 0}^8 {(nu{m_t} \times \mathrm{{code}}Le{n_t}),} }&{0 \le t \le 8} \end{array}} \end{aligned}$$

where $num_t$ is the number of label *t* in the label graph and $\mathrm{{code}}Le{n_t}$ is the length of the codeword corresponding to label *t*.

### Label map embedding

**Figure 7:**
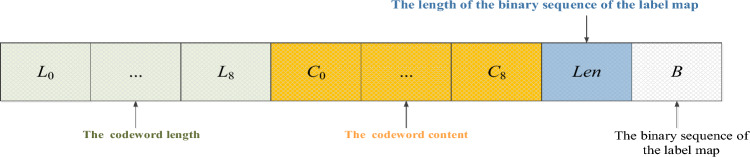
The storage structure of the auxiliary information.

In this section, we take the Huffman encoding rules, the length of the binary sequence of the label map, and the binary sequence of the label map as auxiliary information. The data-hider needs to use this auxiliary information to locate the labels of non-reference pixels in the encrypted image and determine which bits can be embedded into non-reference pixels with different labels. Therefore, it is necessary to embed the auxiliary information in the encrypted image. Figure 7 illustrates the storage structure of this auxiliary information.

From Fig. 7 we know that the variable $L_t$ ($0 \le t \le 8$) is the length of the codeword corresponding to label *t*, where each codeword stores 4 bits in length, $C_t$ is the content of the codeword corresponding to label *t*, and $L_t$ and $C_t$ together form the Huffman coding rules. The parameter *Len* is the length of the binary sequence of the label map, and we use $\left\lceil {lo{g_2}M + lo{g_2}N + 2} \right\rceil$ bits to store *Len*. The last parameter *B* is the binary sequence of the label map. All the stored bit streams form the auxiliary information.

Since the label of the current pixel must be known in advance when extracting the auxiliary information, we store some auxiliary information in the first row and column of the reference pixels. The remaining auxiliary information and the replaced bits of the reference pixels are sequentially embedded in the encrypted image according to Eq. (9).

9 $$\begin{aligned} {{X_{em}}(i,j) = \left\{ \begin{array}{l} \begin{array}{*{20}{c}} {{X_e}(i,j)\bmod {2^{7 - t}} + \sum \limits _{s = 0}^t {{b_s} \times {2^{7 - s}}},}&{}{0 \le t \le 6} \end{array}\\ \begin{array}{*{20}{c}} {\sum \limits _{s = 1}^8 {{b_s} \times {2^{8 - s}}},}&{}{7 \le t \le 8} \end{array} \end{array} \right. } \end{aligned}$$

Where ${X_{em}}(i,j)$ is the encrypted and embedded pixel at position (*i*, *j*), ${b_s}$ is the data to be embedded and *t* is the corresponding label value of the pixel.

### Data embedding

To embed the hidden data, the embeddable bits of each non-reference pixel must be known. The data-hider extracts the auxiliary information stored in the first row and column to obtain the Huffman encoding rules, the length of the binary sequence of the label map and the labels of some non-reference pixels. The label *t* of the current pixel is known, so that $t + 1$ bits of auxiliary information can be extracted ($t \ne 8$, if $t = 8$ then only 8 bits can be extracted). Similarly, all auxiliary information can be extracted from the remaining non-reference pixels in a left-to-right, top-to-bottom order to obtain a complete label map.

Since the lengths of the embedded auxiliary information and reference pixels are known, it is easy to find the starting position for embedding the secret data. Before embedding the secret data, we encrypt it using the stream cipher method. Then, according to the label map, the encrypted secret data can be further embedded into the non-reference pixels of the encrypted image according to Eq. (9).

Let ${L_t}$ be the length of the codeword corresponding to the label *t*, $N_t$ be the number of the label *t* in the label map, $L_m$ be the length of the label map converted into a binary sequence, then the total embedding capacity $C_{total}$, net embedding capacity $C_{net}$, and embedding rate *r* are calculated as follows:

10 $$\begin{aligned} {C_{total}}= & {} \sum \limits _{i = 0}^6 {{N_i} \times (i + 1)} + \sum \limits _{i = 7}^8 {{N_i} \times 8} \end{aligned}$$

11 $$\begin{aligned} {C_{net}}= & {} {C_{total}} - 4 \times 9 - \sum \limits _{i = 0}^8 {{L_i}} - \left\lceil \llceil {lo{g_2}M + lo{g_2}N + 2} \right\rceil \rrceil - {L_m} \end{aligned}$$

12 $$\begin{aligned} r= & {} \frac{{C_{net}}}{{M \times N}} \end{aligned}$$

### Data extraction and image recovery

Before extracting the secret data and recovering the original image, the receiver should extract the auxiliary information from the first row and column. Based on the extracted auxiliary information, the Huffman encoding rules, the length of the binary sequence of the label map and the partial binary sequence of the label map are determined. The receiver can then sequentially extract the embedded data from the non-reference pixels according to the obtained label map.

If the receiver has only the data embedding key $K_h$, the encrypted secret data can be decrypted directly according to the data embedding key $K_h$, and the decryption process is the same as the encryption process. However, without the encryption key $K_e$, the original image cannot be recovered.

If the receiver has only the encryption key $K_e$, the original image can be recovered without any loss. This is because the data hiding key only encrypts the embedded data and does not affect the image processing process. First, the received image is decrypted by $K_e$, and the decryption process is the same as the encryption process. Then the first $t + 1$ bits of each pixel, except the reference pixels, are different from the original pixel, because these pixels are embedded with extra $t + 1$ bits based on their label value *t*. The embedded bits of the non-reference pixels can be recovered from the label map and the predicted values, because the first *t*-MSB of the original pixel is the same as its predicted value, while the ${(t + 1)^{th}}$ MSB of the original pixel can be obtained by negating the ${(t + 1)^{th}}$ MSB of its predicted value. Therefore, the predicted values of non-reference pixels can be obtained by simply using the same prediction method as in Section 3.1 and making predictions based on the first row and column of the recovered reference pixels. In combination with the label map, the original image can be recovered losslessly.

Assuming that the pixel to be recovered is ${X'_{ew}}(i,j)$ and the pixel recovery process is formulated in Eq. (13).

13 $$\begin{aligned} {X(i,j) = \left\{ \begin{array}{l} \begin{array}{*{20}{c}} {{P_X}{{(i,j)}^{tMSB}} + \left( {{P_X}{{(i,j)}^{t + 1}} \oplus 1} \right) \times {2^{7 - t}} + {{X'}_{ew}}(i,j)\bmod {2^{7 - t}},}&{}{0 \le t \le 7} \end{array}\\ \begin{array}{*{20}{c}} {{P_X}(i,j),}&{}{t = 8} \end{array} \end{array} \right. } \end{aligned}$$

Where ${P_X}{(i,j)^{tMSB}}$ is the decimal value of the high MSB bit of the predicted value, which can be calculated by

14 $$\begin{aligned} {{P_X}{(i,j)^{tMSB}} = \sum \limits _{k = 1}^t {{P_X}{{(i,j)}^k} \times {2^{8 - k}}}} \end{aligned}$$

Only with both the data hiding key $K_h$ and the image encryption key $K_e$ can the receiver reversibly extract the secret data and recover the original image.

## Experiments and Analysis

**Figure 8:**
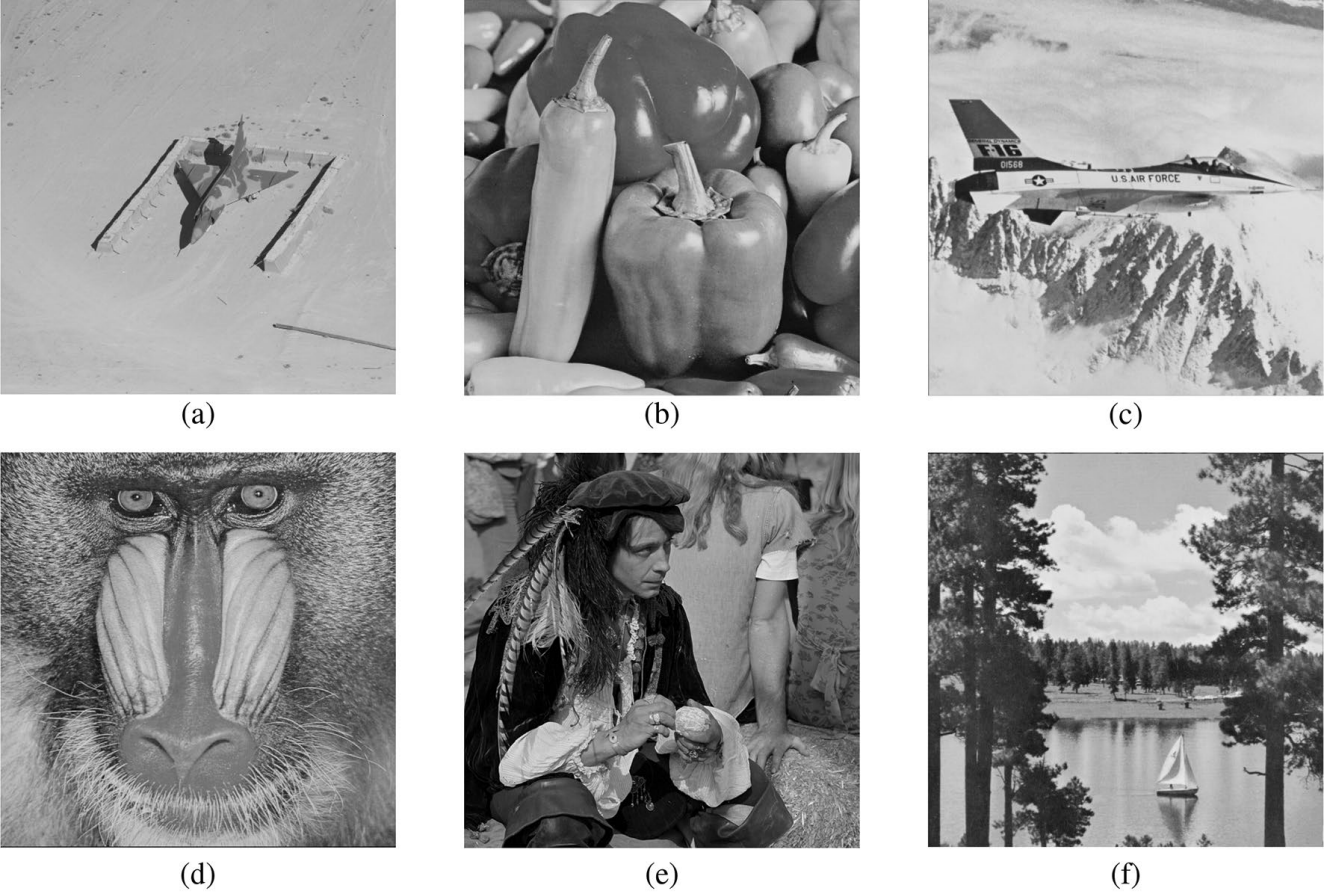
Test images. (**a**) Airplane; (**b**) Peppers; (**c**) Jetplane; (**d**) Baboon; (**e**) Man; (**f**) Lake.

To verify the effectiveness of the proposed algorithm, we conduct a wide range of experimental tests on six grey-scale images^33^, including Jetplane, Peppers, Airplane, Baboon, Man, and Lake (Note that six images are randomly selected for testing purposes, anyone can read the Copyright Information for these images at http://sipi.usc.edu/database/copyright.php), as shown in Fig. 8. In addition, without loss of generality, we test the generalizability of the proposed method on three publicly available datasets BOSSBase^34^, BOWS-2^35^, and UCID^36^, which contain 10,000 images, 10,000 images, and 1,338 images, respectively. In our experiments, since the prediction thresholds for GED and GAP have been experimentally proven in^37^, we set them as follows: ${T_1} = 28$, ${T_2} = 80$, ${T_3} = 32$ and ${T_4} = 8$. The peak signal-to-noise ratio (PSNR) and structural similarity metric (SSIM) were used to evaluate the visual quality of the images. We evaluate the performance mainly in terms of security performance, compression performance and prediction performance. The security performance is assessed using classical statistical correlation analysis, the compression performance is assessed by describing the detailed encoding of the different test images, and the prediction performance is assessed based on a comparison of metrics between different prediction methods.

### The security performance

In contrast to most common encryption methods based on image block encryption, we encrypt each pixel of the original image with a stream cipher, i.e., a pixel-based encryption, which provides a certain level of encryption security. To confirm the security of the proposed method, we will analyze it in terms of statistical features.

**Figure 9:**
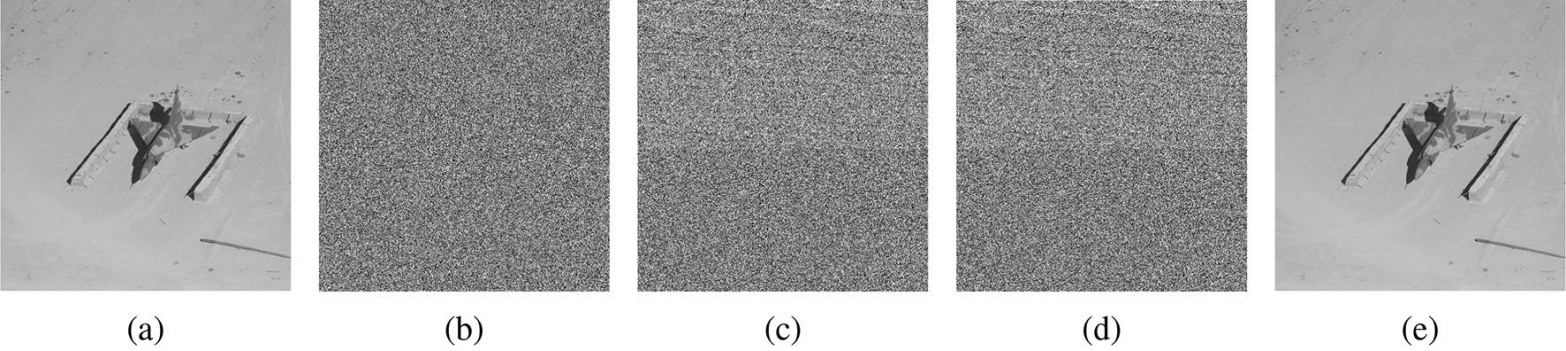
The experiment results of the proposed method. (**a**) Original image; (**b**) Encrypted image; (**c**) Encrypted image with label map; (**d**) Marked encrypted image with net embeddimg rate $ER = 2.7718$; (**e**) Recovered image with $PSNR = \infty , SSIM = 1$.

Taking the image Lena as an example, Fig. 9 shows the experimental results of each stage of the proposed method. From Fig. 9b we can see that the encryption process corrupts the pixel distribution of the original image, making it difficult for an attacker to detect valid plaintext from the encrypted image with the naked eye. To ensure that the data hider can reversibly embed the secret data, we embed the compressed label map into the encrypted image to obtain the encrypted image with label map, which can be seen in Fig. 9c. It can be seen that there is no significant difference between Fig. 9b and c, which means that the embedding of the label map has little effect on the security of the encrypted image. Figure 9d shows the result of the final marked encrypted image. It is easy to see that the proposed method is able to achieve a net embedding rate of $ER = 2.7718$ bit per pixel (bpp) while still ensuring the embedding of the label map. Based on the PSNR and SSIM values of the recovered results shown in Fig. 9e, it can be concluded that the proposed method achieves full reversibility.

**Figure 10:**
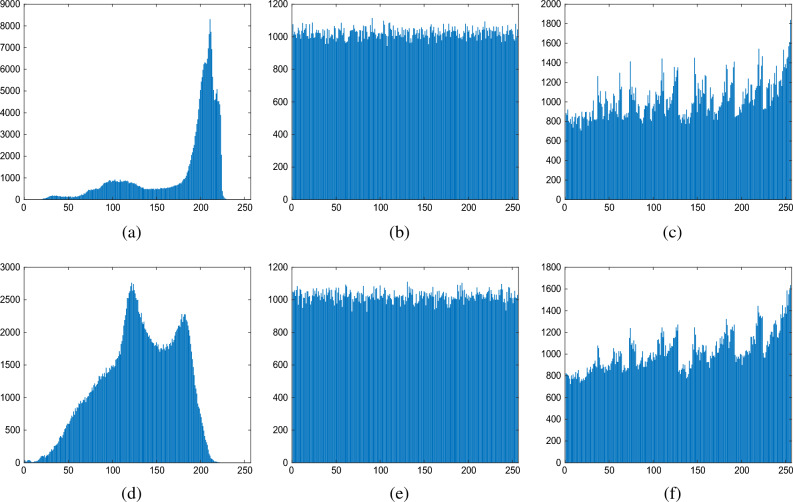
Histograms of three different images for three phases. (**a**–**c**) The histograms of original image, encrypted image and marked encrypted image for Jetplane; (**d**–**f**) The histograms of original image, encrypted image and marked encrypted image for Baboon.

Histogram analysis is a statistical method used to analyze the distribution of pixel frequencies in an image. It can be used to assess the security of an encryption algorithm by comparing the frequency histograms of an image before and after encryption. Generally, the histogram of an encrypted image should exhibit more uniformity and randomness compared to that of the original image. In our statistical analysis experiments, we have chosen Jetplane and Baboon as examples to test the histogram results at different stages. Figure 10 shows the histograms of the original image, encrypted image, and marked encrypted image. From Fig. 10, it is evident that for the original image, pixel distribution is more concentrated due to important features present in it. However, for the histogram of the encrypted image, pixel distribution becomes more uniform and lacks any useful statistical features. The uniform distribution in encrypted images indicates that pixels are modified randomly during encryption process, resulting in slight fluctuations in gray-level values ranging from 0-255. After embedding label maps and secret data into encrypted images, there is a significant increase in individual pixels; however, overall pixel distribution remains largely unchanged. Since encrypted pixels are modified based on Huffman codewords corresponding to labels, histogram distributions of marked encrypted images are not completely uniform like those obtained through pseudo-random key modification for original pixels. In the first and last columns of Fig. 10, it can be clearly seen that there is a noticeable difference in pixel distribution between original images and marked encrypted images. This difference indicates enhanced security for marked encrypted images.

### The compression performance

In this section, we focus on analyzing the compression process of the proposed adaptive Huffman coding using MED+GAP prediction. Tables 1 and 2 show the details of the Huffman compression process based on MED+GAP for the images Lena, Jetplane and Baboon. The value -1 represents the label value of the reference pixel that is not involved in the encoding, while the other 0-8 different types of label values represent non-reference pixels used to embed the secret data; rows 2 and 3 show the number of corresponding label values and the result of the probability distribution; row 4 shows the capacity that can be embedded in each label value; row 5 calculates the total capacity; rows 6 and 7 show the codeword and codeword length of the corresponding label values; and row 8 calculates the length of the binary sequence using adaptive Huffman encoding.

As shown in Tables 1 and 2, once the label values for non-reference pixels have been established, the total capacity of an image is established accordingly. In order to reversibly embed secret data, it is necessary to losslessly compress 0-8 different types of label values, so the choice of a suitable compression method becomes crucial. Based on the data in rows 1 and 2, we can know the probability distribution of the 0-8 different label values, which provides sufficient conditions for the subsequent adaptive Huffman coding. We can construct the Huffman tree by assigning longer codewords to low probability labels and shorter codewords to high probability labels, as shown in line 6. Based on adaptive Huffman coding, the optimal codewords and code lengths can be assigned to different labels of the image. After encoding, the labels of different images are adaptively encoded, and the total capacity minus the length of the label map is taken as the maximum net capacity that secret data can be embedded.

**Table 1:** Huffman compression process with MED+GAP for sized $512 \times 512$ Jetplane.

Label *t*	− 1	0	1	2	3	4	5	6	7	8
Number	1023	4998	10104	10010	24925	34307	45338	46503	36816	48120
Frequency	–	1.9141%	3.8695%	3.8335%	9.5454%	13.1384%	17.3628%	17.8090%	14.0992%	18.4282%
Capacity	–	1	2	3	4	5	6	7	8	8
Total capacity	$4998 \times 1 + 10104 \times 2 + 10010 \times 3 + 24925 \times 4 + 34307 \times 5 + 45338 \times 6 + 46503 \times 7 + 36816 \times 8 + 48120 \times 8 = 1603508$									
Codeword	–	01110	0110	01111	010	100	110	111	101	00
Codeword length	–	5	4	5	3	3	3	3	3	2
Label map length	$4998 \times 5 + 10104 \times 4 + 10010 \times 5 + 24925 \times 3 + 34307 \times 3 + 45338 \times 3 + 46503 \times 3 + 36816 \times 3 + 48120 \times 2 = 775363$

**Table 2:** Huffman compression process with MED+GAP for sized $512 \times 512$ Baboon.

Label *t*	− 1	0	1	2	3	4	5	6	7	8
Number	1023	35666	21130	41142	48478	45444	31802	18292	9451	9716
Frequency	–	13.6588%	8.0920%	15.7559%	18.5653%	17.4034%	12.1790%	7.0052%	3.6194%	3.7209%
Capacity	–	1	2	3	4	5	6	7	8	8
Total capacity	$35666 \times 1 + 21130 \times 2 + 41142 \times 3 + 48478 \times 4 + 45444 \times 5 + 31802 \times 6 + 18292 \times 7 + 9451 \times 8 + 9716 \times 8 = 1094676$									
Codeword	–	100	010	110	00	111	011	1010	10110	10111
Codeword length	–	3	3	3	2	3	3	4	5	5
Label map length	$35666 \times 3 + 21130 \times 3 + 41142 \times 3 + 48478 \times 2 + 45444 \times 3 + 31802 \times 3 + 18292 \times 4 + 9451 \times 5 + 9716 \times 5 = 791511$									

In addition, Table 3 shows the Huffman codewords for six different standard test images. It is evident that for nine labels of different images, the Huffman tree of the proposed algorithm adaptively allocates the optimal codeword to the corresponding label value. With such encoding rules, it is possible to maximize the compression of the label values for different images, and thus to guarantee the amount of secret data that can be embedded.

**Table 3:** Huffman codewords of six standard test images.

Images	Huffman codewords
Airplane	00,01,100,101,110,1111,11101,111000,1111001
Peppers	00,01,100,101,1101,1110,1111,11000,11001
Jetplane	00,010,100,101,110,111,0110,01110,01111
Baboon	00,010,011,100,110,111,1010,10110,10111
Man	00,01,100,101,1101,1110,1111,11000,11001
Lake	01,000,100,110,111,0010,0011,1010,1011

### The prediction performance

In this subsection, we present the main factors that affect the net embedding capacity in an easy to understand formula. This explains why we try to take different prediction methods into account. Assuming a total embedding capacity denoted by $C_{total}$, a net embedding capacity by $C_{net}$, a label map length by $C_{label}$, and an extra bit length by $C_{extra}$, the net embedding capacity can be expressed as:

15 $$\begin{aligned} {{C_{net}} = {C_{total}} - {C_{label}} - {C_{extra}}} \end{aligned}$$

According to Eq. (15), there are three possible ways to increase the net embedding capacity: 1) to increase the total embedding capacity ${C_{total}}$; 2) to decrease the length of the label map ${C_{extra}}$; and 3) to decrease the length of the extra bit ${C_{extra}}$. The features for a given image content are determined by its own structure, so a viable way to increase the overall embedding capacity ${C_{total}}$ is to fully exploit the determined features and to choose the suitable prediction method to generate the label map. This can be verified from the calculation of the overall embedding capacity in Tables 1, 2, 3. Shortening the length of the label map ${C_{label}}$ refers to making the length of the encoded label map as short as possible by choosing a feasible encoding, which is described in detail in the adaptive Huffman encoding process in the previous subsection. The reduction of the extra bit length ${C_{extra}}$ has little effect on the net embedding capacity ${C_{net}}$, since the extra bit length mainly stores information about the length of the nine codewords and the length of the label map. We will verify this elaboration in the next experiments. Based on the above discussion, a reasonable prediction is essential for the improvement of the net embedding capacity.

**Table 4:** The total embedding capacity for different predictions.

Images	Non-adaptive	Adaptive	Adaptive	Adaptive	Adaptive	Adaptive
	MED	MED	GED	GAP	MED+GED	MED+GAP
Airplane	**1659203**	1659203	1647953	1622706	1654453	1632560
Peppers	1351227	1351227	1417491	1397340	**1422673**	1405283
Jetplane	1587880	1587880	1572320	1594417	1577988	**1603508**
Baboon	1074384	1074384	1077438	1088812	1081171	**1094676**
Man	5584907	5584907	5616418	5680845	5627776	**5697691**
Lake	1282281	1282281	1323103	1311452	**1327841**	1318496

Bold values represent the improvements or the optimal performance compared with the related methods.

The total embedding capacity for different predictions is shown in Table 4. From columns 2 and 3 in Table 4, it can be seen that when MED is used for prediction, the total embedding capacity obtained is constant regardless of whether adaptive or non-adaptive encoding is used. This means that the total embedding capacity is not directly related to the selection of the encoding method. From columns 3-5 in Table 4, it can be seen that selecting different prediction methods has a direct effect on the total embedding capacity when the encoding method is fixed. Furthermore, the MED+GAP prediction method achieves the best embedding performance for the same test image. Thus, we can conclude that an appropriate prediction method is important to improve the total embedding capacity.

**Table 5:** The length of label map for different predictions.

Images	Non-adaptive	Adaptive	Adaptive	Adaptive	Adaptive	Adaptive
	MED	MED	GED	GAP	MED+GED	MED+GAP
Airplane	682803	**657136**	739443	738189	742242	742604
Peppers	777928	777928	777515	**770083**	780552	774688
Jetplane	793441	778244	779687	**770738**	782870	775363
Baboon	794941	**786599**	787152	786938	790205	791511
Man	3121506	3121506	3142971	**3106928**	3148960	3115866
Lake	787518	786014	793507	**785631**	796624	790342

Bold values represent the improvements or the optimal performance compared with the related methods.

The length of the label map for the different predictions is given in Table 5. As a comparison with columns 2, 3 in Table 4, we can see from columns 2, 3 in Table 5 that although the encoding method cannot change the total embedding capacity, it directly affects the length of the labelled data. In other words, the selection of encoding method affects the net embedding capacity. For the same test image, a shorter label map length means that more secret data can be embedded. Based on this fact, we can know that the label map length generated by the GAP-based prediction method is shorter overall. This means that more pixels involved in the prediction can produce prediction values that are closer to the current value.

**Table 6:** The length of extra bit for different predictions.

Images	Non-adaptive	Adaptive	Adaptive	Adaptive	Adaptive	Adaptive
	MED	MED	GED	GAP	MED+GED	MED+GAP
Airplane	52	95	90	90	90	90
Peppers	52	88	88	88	88	88
Jetplane	52	87	87	87	87	87
Baboon	52	87	87	87	87	87
Man	54	90	90	90	90	90
Lake	52	87	86	86	86	86

The length of the extra bit under different predictions is given in Table 6. As can be seen from Table 6, the overall length of the extra bit is negligible compared to the length of the label map, and therefore its impact on the overall embedding performance is almost negligible. Finally, the net embedding capacity and embedding rate for different predictions are shown in Table 7. It can be seen that the MED+GAP prediction yields better embedding performance except for Airplane and Peppers. From the analysis of the experimental data at different stages in Tables 4, 5, 6, 7, we can conclude that the selection of the prediction method is crucial for the improvement of the net embedding capacity.

**Table 7:** The net embedding capacity and embedding rate for different predictions.

Images	Non-adaptive	Adaptive	Adaptive	Adaptive	Adaptive	Adaptive
	MED	MED	GED	GAP	MED+GED	MED+GAP
Airplane	**{976348,3.7245}**	{1001972,3.8222}	{908420,3.4653}	{884427,3.3738}	{912121,3.4795}	{889866,3.3946}
Peppers	{573247,2.1868}	{573211,2.1866}	{639888,2.441}	{627169,2.3925}	**{642033,2.4492}**	{630507,2.4052}
Jetplane	{794387,3.0303}	{809549,3.0882}	{792546,3.0233}	{823592,3.1418}	{795031,3.0328}	**{828058,3.1588}**
Baboon	{279391,1.0658}	{287698,1.0975}	{290199,1.107}	{301787,1.1512}	{290879,1.1096}	**{303078,1.1562}**
Man	{2463347, 2.3492}	{2463311, 2.3492}	{2473357, 2.3588}	{2573827, 2.4546}	{2478726, 2.3639}	**{2581735, 2.4621}**
Lake	{494711,1.8872}	{496180,1.8928}	{529510,2.0199}	{525735,2.0055}	{531131,2.0261}	**{528068,2.0144}**

Bold values represent the improvements or the optimal performance compared with the related methods.

### Comparison analysis

Here, the various metrics are compared separately and the impact of the prediction mechanism and the adaptive encoding method on the embedding rate is analyzed. Through the comparative discussion and analysis, we will also provide a step-by-step rationale as to why MED+GAP is ultimately selected as the final prediction solution.

**Figure 11:**
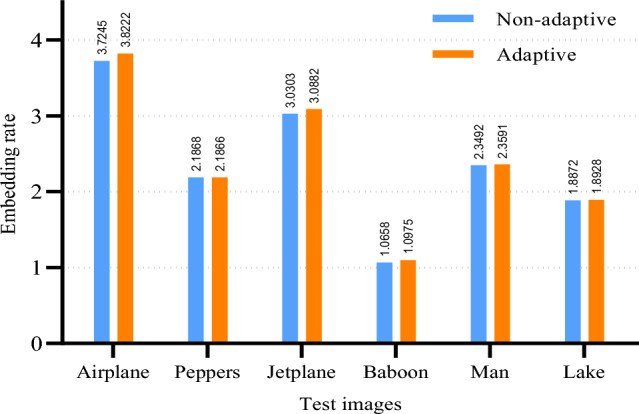
The embedding rate for the non-adaptive and the adaptive encodings based on the MED prediction.

First, Fig. 11 shows the results of the embedding rate for the non-adaptive and the adaptive encoding based on the MED prediction. Note that for the same image, the maximum embedding rate is only calculated with respect to the selected prediction method, so the maximum embedding rate is fixed when the same prediction method is used to generate the label map. The calculation of the maximum embedding rate can be found in Tables 1, 2, 3. According to the results in Fig. 11, the adaptive encoding is able to achieve a higher embedding rate than the non-adaptive encoding. The reason for this is clear from Eq. (16): for a given maximum embedding rate, the shorter the length of the label map, the higher the embedding rate, since the extra bit length has little effect on the embedding rate. Therefore, the reason why adaptive encoding is better than non-adaptive encoding is that adaptive encoding can better adapt to the characteristics of the label map and maximize the compression of the label map data, thus increasing the net embedding capacity, that is, the embedding capacity of the secret data, to a certain extent.

**Figure 12:**
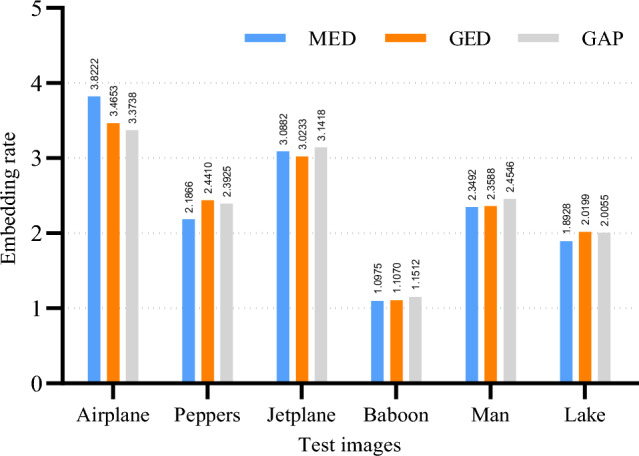
The embedding rate for MED, GED and GAP predictions based on adaptive encoding.

Secondly, Fig. 12 shows the results of the embedding rates obtained using MED, GED and GAP predictions based on the adaptive Huffman encoding. From Fig. 12, we can see that the embedding rates obtained using MED, GED and GAP predictions are slightly different. With the exception of the image Airplane, the embedding rates obtained using the GAP prediction have a better overall performance. This is mainly because GAP uses more neighboring pixels for the prediction of the current pixel. More neighboring pixels means that more local features of the current pixel are captured, making the obtained prediction closer to the current pixel. Based on the results in Fig. 12, we believe that better embedding rates can be achieved by using GAP prediction.

**Figure 13:**
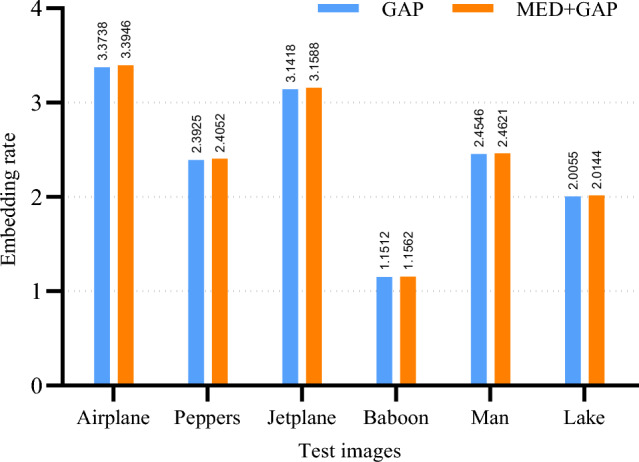
The embedding rate for GAP and MED+GAP predictions based on adaptive encoding.

**Table 8:** Comparison of the net embedding rate for different methods with respect to BOSSBase, BOWS-2, and UCID datasets.

	Datasets	^28^	^31^	^32^	Proposed	Increment
Best case	BOSSBase	2.988	2.988	5.898	6.857	**0.959**
	BOWS-2	2.988	2.988	5.622	6.390	**0.768**
	UCID	2.976	2.98	0 5.010	5.327	**0.317**
Worst case	BOSSBase	0.071	0.004	0.664	0.719	**0.055**
	BOWS-2	0.048	0.005	0.628	0.676	**0.048**
	UCID	0	0.004	0.397	0.466	**0.069**
Average	BOSSBase	2.561	2.553	3.361	3.413	**0.052**
	BOWS-2	2.519	2.521	3.246	3.269	**0.023**
	UCID	2.268	2.151	2.688	2.735	**0.047**

Bold values represent the improvements or the optimal performance compared with the related methods.

So far, the optimal embedding performance can be achieved by the combination of adaptive Huffman encoding and GAP prediction. To further analyze the influence of the prediction, the embedding rate results for the GAP and MED+GAP predictions based on adaptive encoding are presented. The results are shown in Fig. 13. We can see that the embedding rate of the MED+GAP prediction mechanism is superior to that of the GAP prediction. This indicates that more accurate label map data can be generated by using MED to predict the boundaries of non-reference pixels in combination with GAP to predict the remaining non-reference pixels. Therefore, the proposed method selects adaptive Huffman coding and MED+GAP prediction to improve the embedding capacity.

Finally, we use three public datasets, BOSSBase, BOWS-2, and UCID, to compare the net embedding rate for the proposed MED+GAP method with recent related state-of-the-art methods^28,31,32^. Table 8 shows the comparison of the net embedding rates for the three cases. It includes the best embedding rate, the worst embedding rate, and the average embedding rate for different datasets. It can be seen that for the best case scenario, compared with other methods the proposed method provides an increment of 0.959 bpp for BOSSBase dataset, 0.768 bpp for BOWS-2 dataset, and 0.317 bpp , for UCID dataset respectively. Even in the worst case scenario there is still an improvement of 0.055 bpp for BOSSBase dataset, 0.048 bpp for BOWS-2 dataset, and 0.069 bpp for UCID dataset respectively. This indicates that our proposed method can enhance both upper and lower bounds of embedding rates across different datasets. Furthermore, on average our method improves embedding rates by 0.052 bpp and 0.023 bpp on BOSSBase (with a dataset size of 10,000 images) and BOWS-2 (with a dataset size of 10,000 images) respectively; while it achieves an average improvement of 0.047 bpp on UCID (with a dataset size of1 ,338 images). These experimental results demonstrate that our proposed method consistently enhances overall performance in terms of embedding rates.

## Conclusions and future work

In this paper, a novel RDHEI algorithm based on multi-prediction and adaptive Huffman encoding is implemented. The multi-MSBs of non-reference pixels are predicted by median edge detector (MED) and gradient adjusted predictor (GAP). After obtaining the pixel prediction, the same bits of the original and predicted pixels are labelled with adaptive Huffman coding from the highest to the lowest bits. The image is encrypted using a stream cipher method, and secret data is embedded in the free space by multi-MSB substitution. Experimental results demonstrate that this method with MED+GAP prediction and adaptive Huffman coding improves 0.052 bpp, 0.023 bpp, and 0.047 bpp on average over other state-of-the-art methods on the BOSSBase, BOWS-2, and UCID datasets, respectively, while maintaining security and reversibility. Although our method aims to enhance embedding performance by utilizing multi-prediction and adaptive Huffman encoding, it also results in increased time consumption for reserved space due to the introduction of more complex encoding and prediction techniques. This places a greater burden on content owners. Our future work will concentrate on improving time efficiency and further enhancing embedding capacity.

## Data Availability

All data generated or analysed during this study are included in this article.
